# Percutaneous closure versus surgical repair for ruptured sinus of valsalva aneurysm: A systematic review and meta-analysis

**DOI:** 10.3389/fcvm.2023.1158906

**Published:** 2023-04-17

**Authors:** Yong Mao, Cingting Wang, Yongnan Li, Xinqiang Guan, Xiaopeng Zhang, Xiangyang Wu

**Affiliations:** ^1^ Department of Cardiac Surgery, Lanzhou University Second Hospital, Lanzhou University, Lanzhou, China; ^2^ Health Science Center of Lanzhou University, Lanzhou University, Lanzhou, China; ^3^ Dongguan Tungwah Hospital, Dongguan, China

**Keywords:** percutaneous closure, surgical repair, ruptured sinus of valsalva aneurysm, meta-analysis, effects of treatment

## Abstract

**Objectives:**

Ruptured sinus of Valsalva aneurysm (RSVA) often has an abrupt onset, and can chest pain, acute heart failure, and even sudden death. The effectiveness of different treatment modalities remains controversial. Thus, we completed a meta-analysis to evaluate the efficiency and safety of traditional surgery vs. percutaneous closure (PC) for RSVA.

**Methods:**

We carried out a meta-analysis using PubMed, Embase, Web of Science, Cochrane Library, China National Knowledge Infrastructure (CNKI), WanFang Data, and the China Science and Technology Journal Database. The primary outcome was comparing in-hospital mortality between the two procedures, and the secondary outcome was documenting postoperative residual shunts, postoperative aortic regurgitation, and length of hospital stay in the two groups. Differences were expressed as odds ratios (ORs) with 95% confidence intervals (CIs) to assess the relationships between predefined surgical variables and clinical outcomes. This meta-analysis was conducted using Review Manager software (version 5.3).

**Results:**

The final qualifying studies included 330 patients from 10 trials (123 in the percutaneous closure group, and 207 in the surgical repair group). When PC was compared to surgical repair, there were no statistically significant differences in in-hospital mortality (overall OR: 0.47, 95%CI 0.05–4.31, *P* = 0.50). However, percutaneous closure did significantly decrease the average length of hospital stay (OR: −2.13, 95% CI −3.05 to −1.20, *P* < 0.00001) when compared to surgical repair, but there were no significant between-group differences in the rates of postoperative residual shunts (overall OR: 1.54, 95%CI 0.55–4.34, *P* = 0.41) or postoperative aortic regurgitation (overall OR: 1.54, 95%CI 0.51–4.68, *P* = 0.45).

**Conclusion:**

PC may become a valuable alternative to surgical repair for RSVA.

## Introduction

Sinus of Valsalva aneurysm is an uncommon aberration that is typically brought on by a congenital lack of elastic and muscular tissue in the aorta wall or acquired as a result of periaortic inflammation, atherosclerosis, trauma, and/or aortic dissection. Patients who have undergone corrective surgery for congenital heart conditions may also experience it (1). In Eastern populations, rupture incidence is higher in patients who are adolescents or older (2). Ruptured sinus of Valsalva aneurysm (RSVA) necessitate immediate medical attention and can cause rapid heart failure, cardiac tamponade, hemodynamic compromise, and possibly sudden cardiac death (3).

Surgical correction is widely used to treat RSVA, and has low death rates. Unfortunately, it may also increase the risks of incisional infections, post-pericardiotomy syndrome, and scar tissue formation (4). Several different devices have been used to treat RSVA, but occluders have received the most attention and have demonstrated positive outcomes in single-case reports and short-case series (5). However, relatively few studies have compared clinical outcomes between surgical and percutaneous closure (PC) for RSVA. Thus, we conducted a meta-analysis to compare clinical results of PC and surgical repair amongst RSVA patients.

## Material and methods

The components of this meta-analysis were reported using the Preferred Reporting Items for Systematic Reviews and Meta-Analyses (PRISMA) statement (6). The research protocol has also been submitted to the International Platform of Registered Systematic Review and Meta-analysis Protocols (INPLASY2022110131).

## Search strategy

The following seven electronic databases were comprehensively searched: WanFang Data, China Science and Technology Journal Database, China national knowledge infrastructure (CKNI), Web of Science, Cochrane Library, PubMed, and Embase. There were no restrictions set on the language or date of the literature search. The searches began on November 30, 2022. Studies detailed the results of patients over the age of 18 who underwent PC surgery or surgical repair for RSVA. The search was developed based on the PICOS method, and the search terms were (“Ruptured sinus of valsalva aneurysm” OR “Sinus of Valsalva aneurysm”) AND (“surgery” OR“percutaneous closure surgery” OR “treatment”). We also manually searched reference lists of retrieved publications (including reviews) to find studies that might be eligible.

## Study selection and inclusion criteria

After deleting duplicates and importing all citations into EndNote, two reviewers (YM and XYW) evaluated the titles and abstracts with consideration of the qualifying standards (shown in Table 1). We only included studies written in English. Full papers that were evaluated and determined to be “included” or “uncertain” were then evaluated once again with consideration of the inclusion criteria. Only studies with the most comprehensive data and that had consistently been published were chosen. The papers were selected through discussion, and the disagreements were finally resolved by a third-party reviewer (CTW).

**Table 1:** Meta-analysis inclusion and exclusion criteria.

	Inclusion criteria	Exclusion criteria
Language	English	Not English
Publication dates	All years	/
Participants	– Ages ≥ 18 years old – RSVA Patients	– Ages < 18 years old – Pregnant patients
Intervention	– Surgical repair – Percutaneous closure	Not in line with the inclusion criteria
Study design	– Randomized controlled trial – Case control study – Cohort study	– Case report – Review – Protocol – Commentary – Letter
Outcome	– In-hospital mortality – Postoperative residual shunt – Postoperative aortic regurgitation – Length of hospital stay	– Data about mortality or other outcomes not available

## Data extraction

A specialized extraction form was used to gather information about each study's author(s), publication year, methodological design, control and treatment interventions, sample size, and findings. If more information was needed, the authors were contacted.

## Quality assessment

The caliber of all included studies was independently assessed by two reviewers (YM and XYW). Nonrandomized control trials were assessed using the Newcastle-Ottawa Scale(NOS) (7). The “star system” was used to grade each included study. A total score of 5 or less was considered low, 6 or 7 was considered moderate, and 8 or 9 was considered high. Disputes were settled by discussion and consensus between the other two reviewers (YNL and CTW). A comparison of in-hospital mortality for the two treatments served as the primary outcome. The secondary outcome was documenting postoperative residual shunts, postoperative aortic regurgitations, and length of hospital stays.

## Statistical analysis

Review Manager 5.3 was used for the meta-analysis and Egger's regression test. For dichotomous variables, the Mantel-Haenszel (MH) model was used to obtain Odds Ratio (OR) and 95% CI. Heterogeneity between studies was assessed by *I*^2^ statistics. *I*^2^ Values of 25, 50, and 75% were reported as low, moderate, and high degrees of heterogeneity, respectively. A subgroup analysis in the meta-analysis (focused on different study designs such as randomized controlled trials, prospective cohort studies, and retrospective studies) was conducted to lessen the heterogeneity. A *P* < 0.05 was considered to be statistically signiﬁcant. Egger's regression model was used to detect publication bias when the number of studies analyzed was enough.

## Results

**Figure 1:**
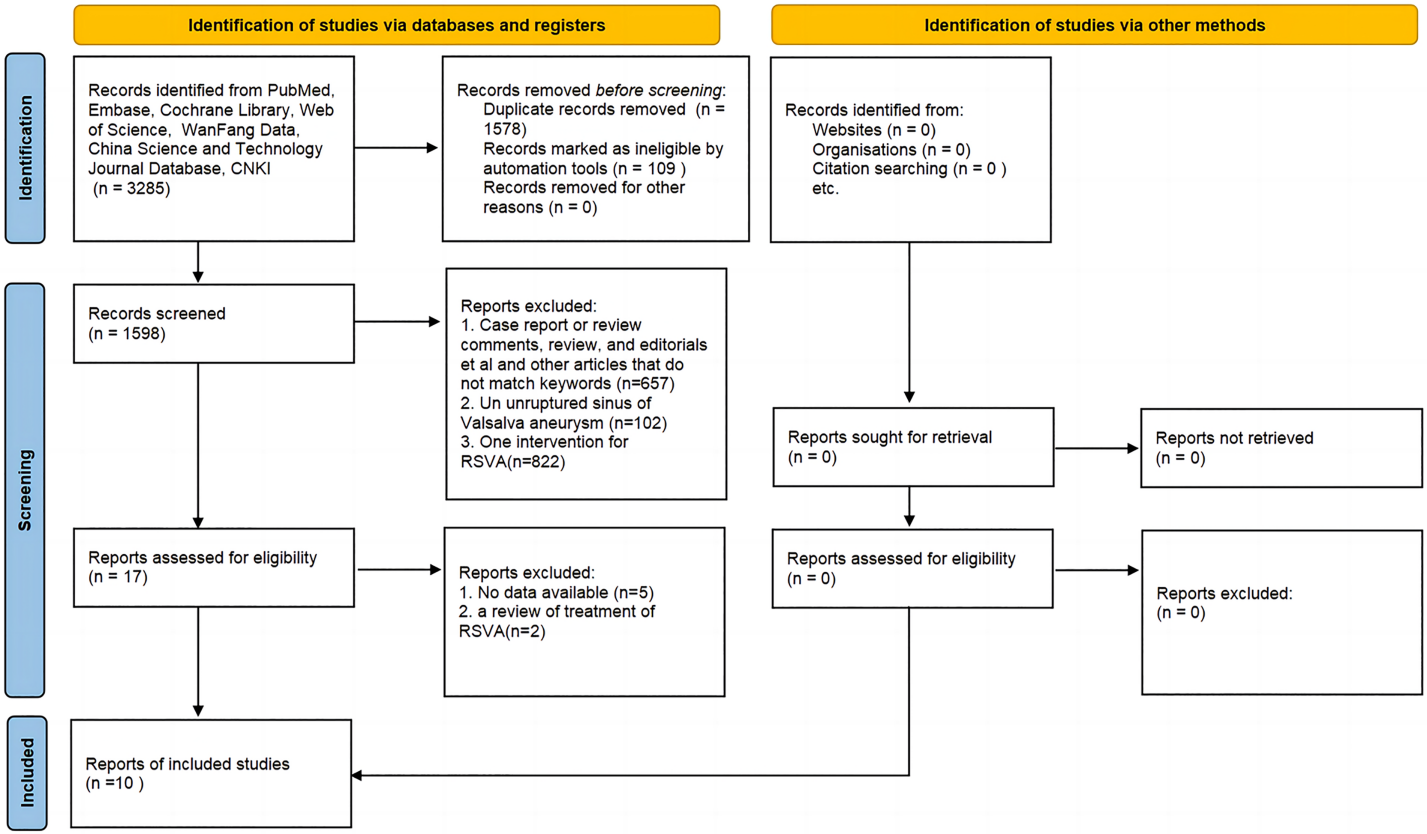
PRISMA flow chart.

A summary of the study selection process is presented in Figure 1. A total of 3,285 citations were found through our literature search. Of these, 1,687 articles were eliminated for various reasons, including duplication. After reviewing the paper titles and abstracts, 1,581 articles were eliminated for PI-VSR or other reasons, depending on the type of article. Six papers were found to be invalid after the full-text versions of 17 publications were reviewed. A total of 10 papers (8–17) were deemed suitable for final analysis.

## General characteristics of the included studies

The key characteristics of the studies that fit the inclusion criteria are presented in Table 2. A total of 330 patients were included across these 10 papers. 123 of them were in the PC group, and 207 were in the surgical repair group. Nine studies compared surgical treatment and PC for RSVA. The other study compared surgical treatment, PC, and medical treatment for RSVA. Nine of the studies evaluated in-hospital mortality, and eight evaluated the rates of residual shunts following surgery, five evaluated rates of postoperative aortic regurgitation, and four evaluated the length of hospital stay.

**Table 2:** Descriptive characteristics of the included studies.

Study	Study type	Age, year	Male, *n*	Patients Number	Intervention Comparison	Defect size, mm	Outcomes	NOS scores
Jiawang et al. 2017 (8)	Retrospective cohort study	37	58	85 (29/56)	PC vs. SR	6.45 vs. 9.18	①②③	8
Supratim et al. 2010 (9)	Retrospective cohort study	29.9	NR	21 (8/13)	PC vs. SR	NR	①	7
Vijayasekaran et al. 2013 (10)	Retrospective cohort study	32	7	13 (4/4/5)	PC vs. SR vs.MT	NR	①③	8
Kai et al. 2020 (11)	Retrospective cohort study	37	30	58 (26/32)	PC vs. SR	7.0 vs. 8.0	①②③④	7
Suxuan et al. 2014 (12)	Retrospective cohort study	42	23	35 (15/20)	PC vs. SR	6.0 vs. 10.0	①②③④	7
Mingtai et al. 2010 (13)	Retrospective cohort study	34	21	35 (6/29)	PC vs. SR	8.0 vs. 10.0	②③	8
Zeeshan et al. 2022 (14)	Retrospective cohort study	37	13	17 (12/5)	PC vs. SR	10.1 vs. 11.0	①②③④	7
Ling et al. 2015 (15)	Retrospective cohort study	48	15	22 (10/12)	PC vs. SR	NR	①②	6
Fengyun et al. 2010 (16)	Retrospective cohort study	35	13	18 (2/16)	PC vs. SR	NR	①②	7
Rui et al. 2018 (17)	Retrospective cohort study	36	18	31 (11/20)	PC vs. SR	9.3 vs. 9.1	①②④	8

SR, surgical repair; PC, percutaneous closure; CT, conservative treatment; ①, in-hospital mortality; ②, postoperative residual shunts; ③, postoperative aortic regurgitation; ④, time of hospital stay.

## Primary outcome

### In-hospital mortality

**Figure 2:**
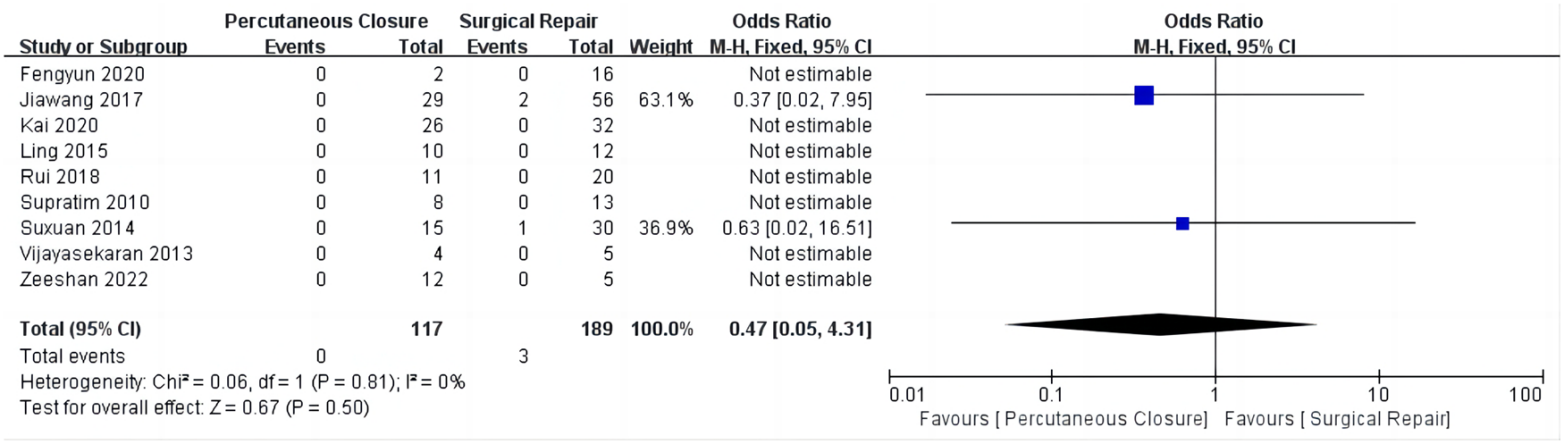
Comparison of in-hospital mortality between percutaneous closure and surgical repair.

There were no significant differences observed in in-hospital mortality rates between the two treatment groups (overall OR: 0.47, 95%CI 0.05–4.31, *P* = 0.50) (Figure 2). Additionally, no heterogeneity was observed (*I*^2^ = 0%).

## Secondary outcomes

### Rates of postoperative residual shunt

**Figure 3:**
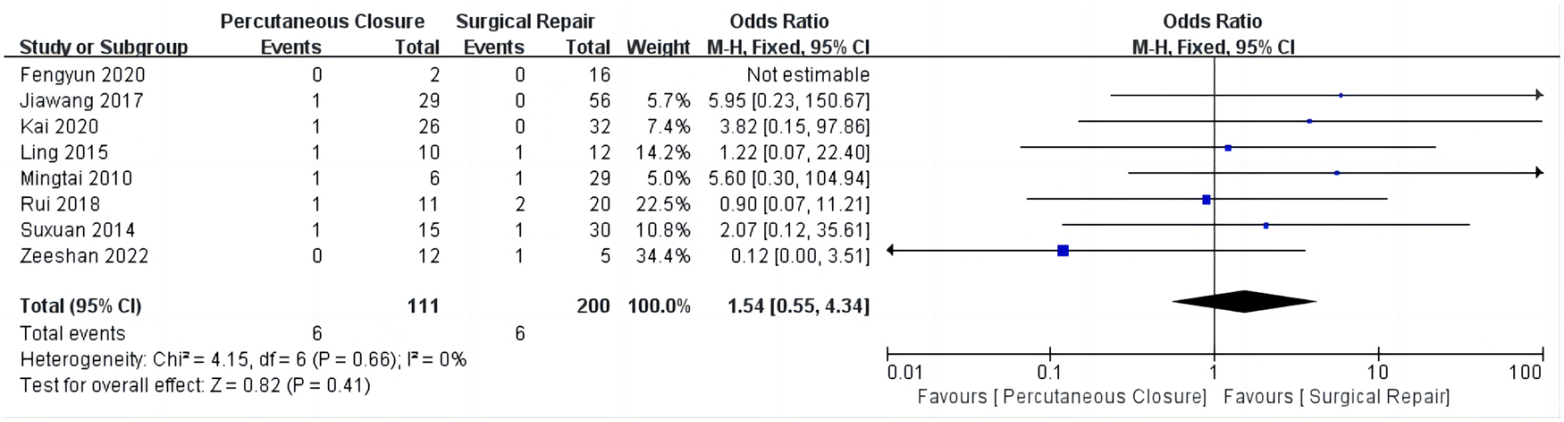
Comparison of postoperative residual shunt rates between percutaneous closure and surgical repair.

There were no significant differences in incidence rates of postoperative residual shunt between the two treatment groups (overall OR: 1.54, 95%CI 0.55–4.34, *P* = 0.41) (Figure 3), and no heterogeneity was observed (*I*^2^ = 0%).

### Rates of postoperative aortic regurgitation

**Figure 4:**
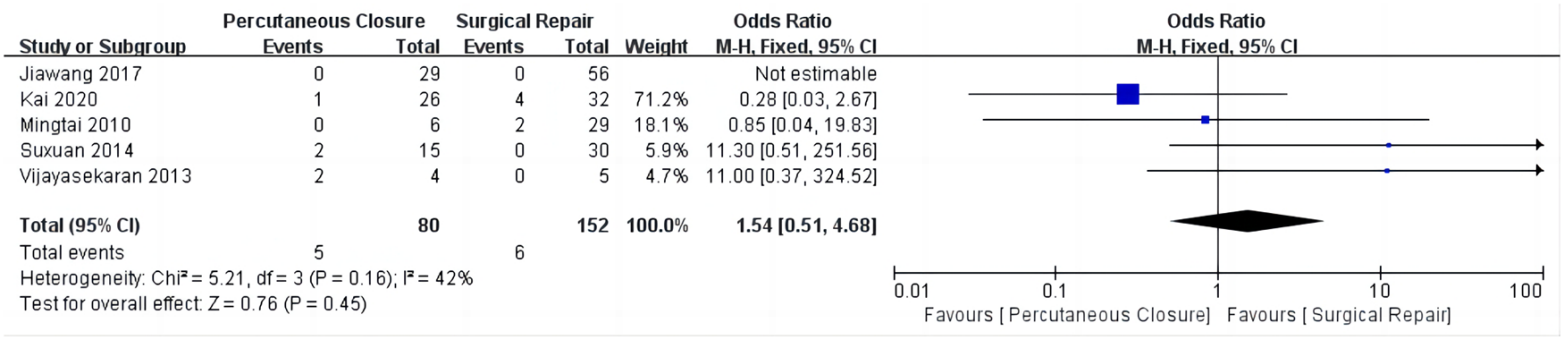
Comparing postoperative aortic regurgitation of percutaneous closure and surgical repair.

There were no significant differences in the incidence rates of postoperative aortic regurgitation between the treatment groups (overall OR: 1.54, 95%CI 0.51–4.68, *P* = 0.45) (Figure 4), and no heterogeneity was observed (*I*^2^ = 0%).

### Length of hospital stay

**Figure 5:**
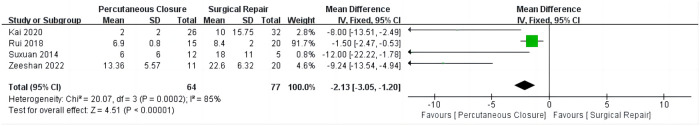
Comparing length of postoperative hospital stay between percutaneous closure and surgical repair.

The PC group had significantly shorter lengths of hospital stay when compared to the surgical treatment group (overall OR: −2.13, 95%CI −3.05–1.20, *P* < 0.00001) (Figure 5). However, high heterogeneity was observed in this category (*I*^2^ = 85%).

## Discussion

Although the actual prevalence of SOVAs is unknown, it is estimated that 0.09 percent of the population is affected. Between 0.1% to 3.5% of all congenital heart abnormalities are SOVA (3). Hemodynamic disturbances and/or sudden cardiac death are frequently present in SOVA patients. There have also been reports of rupture into the interventricular septum, leading to occlusion of the left ventricular outflow system (18). This investigation demonstrated that PC treatment for RSVA was associated with a shorter length of hospital stay compared to surgical treatment. However, there were no statistically significant differences in in-hospital mortality rates, incidence rates of postoperative residual shunts, or incidence rates of postoperative aortic regurgitation between the two strategies.

Surgical treatment is widely used to treat RSVA, especially in patients with combined intracardiac malformations. The literature reports a long history of surgical treatment for RSVA, with the first surgical treatment performed by Lillehei in 1957 (19). Surgical treatment has several advantages, including complete correction of the deformity and maturity of practice. However, surgical treatment can increase other risks and even cause death. Additionally, patients will often opt for an interventional approach if it can achieve the same results as surgery. Cullen et al. described the first instance of PC in 1994, and this less invasive approach has been increasingly used (20). Liu reported on 25 patients from a single center and found that trivial residual shunts developed in three patients, and mild occluder-related AR occurred in five patients. During a median follow-up time period of 19 months (6–96 months), however, all of the trivial residual shunts vanished, and mild occluder-related AR disappeared in four out of the five patients (21). In addition, studies from several centers have shown that interventions can be used to treat PDA, ASD, and VSD in combination with RSVA (12, 22). However, there are still no universal guidelines for RSVA treatment, so we completed a meta-analysis and systematic review of this disease.

The effectiveness of percutaneous intervention and surgical repair have not been previously compared in a meta-analysis. Kuriakose compared 34 studies detailing PC treatments with 16 studies on surgical closure, including 877 patients treated for aortic sinus aneurysm ruptures between 1,956 and 2014. He concluded that PC might be safe, effective, and practical in patients who are too ill for bypass surgery and who have mild or no aortic regurgitation and simple associated defects (e.g.,myxomatous ventricular septal defects, secondary foramen ovale septal defects, and small patent ductus arteriosus) (23). His conclusions are generally consistent with ours. The prolonged length of hospital stay amongst patients in the surgical group in our study may be related to factors such as extracorporeal circulation and the more invasive nature of the procedure.

This meta-analysis had several limitations. First, the included studies were all retrospective, and it was not possible to control for potential confounding factors. In addition, The location of Additionally, the precise location of the ruptures may have differed (see Table 3).

**Table 3:** RSVA locations in the studies.

	Jiawang et al. 2017 (8)		Supratim et al. 2010 (9)		Vijayasekaran et al. 2013 (10)		Kai et al. 2020 (11)		Suxuan et al. 2014 (12)		Mingtai et al. 2010 (13)		Zeeshan et al. 2022 (14)		Ling et al. 2015 (15)		Fengyun et al. 2010 (16)		Rui et al. 2018 (17)	
Location	PC	SR	PC	SR	PC	SR	PC	SR	PC	SR	PC	SR	PC	SR	PC	SR	PC	SR	PC	SR
RCS to RA	7	22	0	2	NR	NR	13	11	4	2	NR	NR	1	1	NR	NR	NR	NR	NR	NR
RCS to RV	15	28	8	6	NR	NR	6	8	5	16	NR	NR	9	4	NR	NR	NR	NR	NR	NR
NCS to RA	7	3	0	4	NR	NR	7	13	5	2	NR	NR	2	0	NR	NR	NR	NR	NR	NR
NCS to RV	0	2	0	1	NR	NR	0	0	1	0	NR	NR	0	0	NR	NR	NR	NR	NR	NR
LCS to LA	0	1	0	0	NR	NR	0	0	0	0	NR	NR	0	0	NR	NR	NR	NR	NR	NR
Total	29	56	8	13			26	32	15	20			12	5						

## Conclusions

RSVA is a rare cardiac malformation that can result in large left-to-right shunts and severe congestive heart failure. We conducted a meta-analysis and concluded that, for RSVA, PC might become a valuable alternative to surgical repair. However, large-scale randomized controlled trials are required to confirm the effects of PC and surgical repair for treating RSVA.
